# Analyzing clinical and genetic characteristics of a cohort with multiple congenital anomalies-hypotonia-seizures syndrome (MCAHS)

**DOI:** 10.1186/s13023-020-01365-0

**Published:** 2020-03-27

**Authors:** Xianru Jiao, Jiao Xue, Pan Gong, Xinhua Bao, Ye Wu, Yuehua Zhang, Yuwu Jiang, Zhixian Yang

**Affiliations:** Department of Pediatrics, Peking University First Hospital, No.1, Xi’anmen Street, Xicheng District, Beijing, 100034 China

**Keywords:** Glycosylphosphatidylinositol anchor, Epilepsy, Hypotonia, Developmental delay, Dysmorphism

## Abstract

**Objective:**

To summarize and extend the phenotypic characterization of Multiple Congenital Anomalies-Hypotonia-Seizures Syndrome, and to discuss genotype-phenotype correlations.

**Methods:**

Collecting clinical information of 17 patients with pathogenic variants in *PIGN*, *PIGA*, and *PIGT.* Genetic studies were performed on all patients.

**Results:**

There were 7 patients with 15 *PIGN* mutations (one patient carrying 3 mutations), 8 patients with 8 *PIGA* mutations, and 2 patients with 5 *PIGT* mutations (one patient carrying 3 mutations). All patients had epilepsy and developmental delay, with 71% of them showed hypotonia. And among these patients’ various seizure types, the focal seizure was the most common one. Eighty-two percent patients showed a significant relationship between seizures and fever. Serum ALP was elevated in one patient with *PIGN* mutations and in two patients with *PIGA* mutations. Brain MRI showed enlarged subarachnoid space in 56% of patients. Some other different characteristics had also been found in our patients: First, atypical absence seizures presented in three patients with *PIGN* mutations; Second, diffuse slow waves mixed with focal or multifocal discharges of interictal EEG in 88% cases with *PIGA*-deficient; Third, phenotypes of seven out of eight patients with *PIGA* mutations were difficult to be classified as severe or less severe group; Last, mild neurological symptoms and developmental status rather than severe conditions occurred in one patient with *PIGT* mutations.

**Conclusion:**

With epilepsy, developmental delay, and/or hypotonia as common features, the knowledge of MCAHS in terms of phenotype and genotype has been expanded. In cases with *PIGN*-deficient, we expanded the types of atypical absence seizures, and described one patient with elevated serum ALP. Focal seizures with diffuse slow waves mixed with focal or multifocal discharges on EEG rather than infantile spasms with hypsarrhythmia, which as previously reported were often seen in our patients with *PIGA* mutations. The classifications of phenotypes caused by *PIGA* mutations should be more continuous than discrete. The mild phenotype of one patient with *PIGT* mutations expanded the clinical presentation of MCAHS3.

## Introduction

Glycosylphosphatidylinositol (GPI) anchoring is a highly conserved process that enables proteins to attach to the cell surface membrane [1, 2]. Close to 30 genes have been identified, that are involved in the biosynthesis and remodeling of GPI-anchored proteins (GPI-APs), and variants in nearly 20 genes are associated with human diseases [3–5]. The function of GPI-related proteins is impaired due to defects in GPI anchored biosynthesis pathway, which have caused broad clinical phenotypes. The Multiple Congenital Anomalies-Hypotonia-Seizures Syndrome (MCAHS) is a clinically and etiologically heterogeneous disorder caused by mutations in the phosphatidylinositol glycan family, featuring hypotonia, seizures, multiple congenital anomalies, and delayed or lacking psychomotor development [5].

In 2012, the homozygous mutations of phosphatidylinositol glycan biosynthesis class N protein (*PIGN*, OMIM: 614080) was initially reported to lead to MCAHS1 in seven patients from a large consanguineous Israeli-Arab family [5]. Then, phosphatidylinositol glycan biosynthesis class A protein (*PIGA*, OMIM: 300868) and phosphatidylinositol glycan biosynthesis class T protein (*PIGT*, OMIM: 615398), in turn, were found to be responsible for MCAHS2 and MCAHS3 [6, 7]. In addition to *PIGA* being an X-linked recessive inheritance, both *PIGN* and *PIGT* were consistent with the characteristics of autosomal recessive inheritance.

*PIGN* controls the addition of phosphoethanolamine to the first mannose in GPI [8]. To date, a total of 29 patients with 22 different variants have been reported [5, 9–19]. Nineteen patients were diagnosed with MCAHS1 and 10 were diagnosed with Fryns syndrome which features were congenital diaphragmatic hernia (CDH), dysmorphic facial features, pulmonary hypoplasia, and other various possible internal malformations [5, 9–19]. CDH and associated pulmonary hypoplasia were responsible for early death in most patients with Fryns syndrome [9, 16, 18]. Because the patients with Fryns syndrome died during pregnancy or within a few days of birth, epilepsy and developmental delay were not observed in these patients [9, 16, 18]. MCAHS1 and Fryns syndrome share overlapping clinical features. The human *PIGA* maps to chromosome Xp22.2. Somatic *PIGA* mutations have been identified in many patients with paroxysmal nocturnal hemoglobinuria [20, 21]. Subsequently, germline *PIGA* mutations were associated with a spectrum of X-linked disorders containing MCAHS2 [6]. Up to now, 25 male patients carrying 15 different *PIGA* variants have been reported [6, 22–34]. *PIGT* encodes transaminase subunits involved in the GPI anchor binding to the protein at step 11 [35]. So far, 29 patients carrying 15 different *PIGT* variants have been reported [7, 36–43]. Since *PIGN*, *PIGA*, and *PIGT* are widely expressed in different tissues, clinical phenotypes of MCAHS are often complex and diverse.

Here, we present the clinical, biochemical and molecular characteristics of 17 new cases with MCAHS, in which the exome sequencing has revealed mutations in *PIGN*, *PIGA*, and *PIGT*.

## Materials and methods

### Ethics statement

This study was approved by the Biomedical Research Ethical Committee of Peking University First Hospital. The individuals’ parents in this manuscript have given the written informed consents to publish the case details.

### Patients

Seventeen patients from 17 unrelated families were recruited. Several clinical data were analyzed. Electroencephalogram (EEG), brain magnetic resonance imaging (MRI), and biochemical studies were performed in all patients. Development assessment was also performed based on intelligence tests (Wechsler or Gesell intelligence scales) or clinical judgment and parents’ questionnaires.

### Genetic analysis

Genetic analysis of *PIGN*, *PIGA*, and *PIGT* was performed as described by Maydan et al., Johnston et al., and Kvarnung et al. [5–7]. We also predicted the functional alteration of the mutations using polymorphism phenotyping-2 (PolyPhen-2, http://genetics.bwh.harvard.edu/pph2/), SIFT (http://sift.jcvi.org/www/SIFT_seq_submit2.html) and Mutation Taster (http://www.mutationtaster.org/).

## Results

### Clinical features of patients with the *PIGN*/*A*/*T* mutations

The clinical information of individuals with *PIGN*, *PIGA*, and *PIGT* mutations is summarized in Table 1.

**Table 1:** Summary of *PIGN*, *PIGA*, and *PIGT* patients

PIGN (NM_012327)								
Patient ID	Patient 1	Patient 2	Patient 3	Patient 4	Patient 5	Patient 6	Patient 7	
Current age/Sex	2 yrs./M	4 yrs. 9 mo/M	3 yrs. 6 mo/M	2 yrs. 8 mo/F	1 yrs. 7mo/M	2 yrs./M	2 yrs. 5 mo/F	
Gestation, wk	full term	full term	full term	36w	full term	full term	full term	
Birth history	normal	normal	normal	rupture of membranes	normal	normal	normal	
Seizure-onset age	3 mo	7 mo	1 yr	4 mo	9 mo	10 mo	8 mo	
Seizure type	FS	FS, PGTCS, MS	GTCS	FS; AA	FS, AA	MS, AA	FS	
Seizure prognosis	intractable	seizure-free at 3 yrs. with AEDs	intractable	intractable	intractable	intractable	intractable	
Ever used pyridoxine/effect	–	+/−	+/−	+/n.a.	+/n.a.	–	–	
Used AEDs	LEV	OXC, VPA, LEV	LEV	VPA, TPM, LEV	VPA	VPA, LEV	VPA, TPM, OXC	
Fever with seizure breakthrough	+	+	+	+	+	+	+	
Developmental delay	normal motor, severe intellectual	severe	normal motor, severe intellectual	severe	severe	moderate	moderate	
Dysmorphism								
Facial	n.a.	arched palatal	nasal bridge collapse, ocular hypertelorism, nasal tip round blunt	ocular hypertelorism, nasal bridge collapse, arched palatal	binocular esotropia; nystagmus	high forehead	nasal bridge collapse	
Other organs	n.a.	perianal abscess, mild liver enlargement	umbilical hernia, increased foot wrinkles	bilateral Achilles tendon contracture, small hands and feet	–	–	–	
Hypotonia	n.a.	**+**	**+**	+	+	–	+	
Serum ALP	**n.a.**	normal	normal	elevated	n.a.	normal	normal	
Initial interictal EEG	normal	left frontal and central regions discharges	normal	multifocal discharges	normal	multifocal discharges	normal	
Brain MRI	enlarged ventricles, enlarged subarachnoid space	normal	enlarged subarachnoid space	enlarged ventricles, thin corpus callosum, enlarged subarachnoid space	enlarged subarachnoid space, slightly delayed myelin sheath	normal	enlarged ventricles, cerebellar vermis dysplasia	
PIGA (NM_002641)								
Patient ID	Patient 8	Patient 9	Patient 10	Patient 11	Patient 12	Patient 13	Patient 14	Patient 15
Current age/Sex	3 yrs. 10 mo/M	3 yrs. 7 mo/M	6 yrs./M	6 yrs./M	7 yrs./M	4 yrs./M	5 yrs./M	2 yrs. 9 mo/M
Gestation, wk	full term	full term	full term	36 + 1w	n.a.	full term	n.a.	full term
Birth history	normal	normal	normal	anoxia	meconium-stained amniotic fluid	normal	normal	normal
Seizure-onset age	2 mo	10 mo	1 mo 28d	6 mo	7 mo	4 mo	6 mo	6 mo
Seizure type	FS	FS, PGTCS, SE	FS, SE	FS, SE	FS, MS, absence seizure	FS, ES	FS, GTCS	FS
Seizure prognosis	intractable	seizure-free at 1.3 yrs. with AEDs	intractable	intractable	intractable	intractable	seizure-free at 2 yrs. with AEDs	intractable
Ever used pyridoxine/effect	–	–	+/−	+/−	–	+/−	–	–
Used AEDs	TPM, VPA, OXC	VPA, LEV, TPM, OXC	VPA, LEV, TPM	VPA, LEV, TPM	VPA, TPM, LEV	TPM, VPA, LEV	VPA, LEV, TPM	TPM, OXC, VPA, LTG, CLB
Fever with seizure breakthrough	+	+	–	+	+	–	+	+
Developmental delay	severe	severe	severe	severe	severe	normal motor, severe intellectual	severe	severe
Dysmorphism								
Facial	–	ocular hypertelorism	ocular hypertelorism, nasal bridge collapse, arched palatal	arched palatal	–	–	–	n.a.
Other organs	–	foot deformity, umbilical hernia	congenital megacolon	congenital megacolon, cafe-au-lait-spot	–	–	–	n.a.
Hypotonia	+	+	+	+	–	–	n.a.	n.a.
Serum ALP	n.a.	n.a.	elevated	normal	n.a.	elevated	n.a.	n.a.
Initial interictal EEG	diffuse slow waves mixed with predominantly posterior discharges	diffuse slow waves mixed with multifocal discharges	diffuse slow waves mixed with bilateral occipital discharges	diffuse slow waves mixed with multifocal discharges	diffuse slow waves mixed with frontal discharge	hypsarrhythmia	diffuse slow waves mixed with left frontal discharge	diffuse slow waves mixed with multifocal discharges
Brain MRI	white-matter immaturity, enlarged subarachnoid space	enlarged ventricle	enlarged subarachnoid space	enlarged subarachnoid space, deeper brain sulcus	normal	enlarged ventricles, enlarged subarachnoid space	normal	n.a.
PIGT (NM_015937)								
Patient ID	Patient 16	Patient 17						
Current age/Sex	13 yrs. 4 mo/M	1 yr/M						
Gestation, wk	full term	full term						
Birth history	normal	normal						
Seizure-onset age	3 yrs.	4 mo						
Seizure type	FS	ES, MS						
Seizure prognosis	intractable	intractable						
Ever used pyridoxine/effect	–	–						
Used AEDs	VPA, LEV	LEV, VPA, OXC						
Fever with seizure breakthrough	+	–						
Developmental delay	mild	severe						
Dysmorphism								
Facial	–	nasal bridge collapse, arched palatal, dysplastic ears						
Other organs	–	cafe-au-lait-spot						
Hypotonia	–	+						
Serum ALP	n.a.	normal						
Initial interictal EEG	left anterior and middle temporal discharges	multifocal discharges						
Brain MRI	normal	enlarged subarachnoid space						

*y* year, *mo* month, *d* day, *F* femal, *M* male, *FS* focal seizure, *PGTCS* partial secondarily generalized tonic-clonic seizures, *MS* myoclonic seizure, *GTCS* generalized tonic-clonic seizure, *AA* atypical absence, *SE* status epilepticus, *ES* epileptic spasm, *AEDs* antiepileptic drugs, *LEV* levetiracetam, *OXC* oxcarbazepine, *VPA* valproic acid, *TPM* topiramate, *LTG* lamotrigine, *CLB* clobazam, *n.a.* not available, *EEG* electroencephalogram, *MRI* magnetic resonance imaging

#### Phenotype of *PIGN* mutation

One patient (patient 4) out of seven patients had a history of preterm birth due to premature rupture of membranes. The other six patients were delivered at full term. The first seizures began between 3 months and 1 year. Focal seizures occurred in five patients (patient 1, 2, 4, 5, and 7), and other seizure types included atypical absence (3/7), myoclonic seizures (2/7), partial secondarily generalized tonic-clonic seizures (PGTCS) (1/7), and generalized tonic-clonic seizures (GTCS) (1/7). For five patients (patient 3, 4, 5, 6, and 7), the first seizures occurred in the course of febrile illness, after which febrile or afebrile seizures developed. Patient 2 had the first seizures during a hot bath, followed by focal seizures occurring about 3–4 times a month, almost always in the course of febrile illness. Although seizures of patient 1 developed without fever, almost all the subsequent seizures appeared during fever period. Facial deformities presented in six patients while other organ malformations were seen in three. Relevant facial and organ features of patient 1 were not available. Except that patient 1 who had no available results, five patients showed hypotonia.

Initial interictal EEG showed multifocal discharges in two patients (case 4 and 6). Patient 2 first showed discharges in left frontal and central regions, and had then been brought under control by the 5-month treatment of multiple antiepileptic drugs (AEDs). Initial interictal EEG of patient 1, 3, 5, and 7 were normal, however with only patient 5 exhibiting generalized discharges in re-examination 1 year later. The brain MRI of four patients showed enlarged subarachnoid space. In addition, other abnormalities, including enlarged ventricles (3/7), thin corpus callosum (1/7), slightly delayed myelin sheath (1/7), and cerebellar vermis dysplasia (1/7), were also present. By contrast, patient 2 and patient 6 showed neither dysmorphic features nor abnormalities in brain MRI. Serum alkaline phosphatase (ALP) was elevated in patient 4 (369 IU/L, normal range: 161.8–296.4 IU/L). The expression levels of total GPI-APs, CD16, CD24, and CD59 in peripheral blood granulocytes of patient 3 (the only patient tested) decreased to 34%, 28.9%, 19.1%, and 61.6% of normal controls, respectively.

In terms of drug treatments, seizures of six patients were refractory to AEDs, while those of patient 2 were controlled by levetiracetam. As for pyridoxine treatment for four patients, patient 2 was first treated orally with pyridoxine (90 mg/day) without effect at the age of one, and then at the aged of three seizures were controlled by AEDs. Also treated with pyridoxine (40 mg/day) combined with levetiracetam at the age of four, the condition of patient 3 failed to be controlled. Both patients 4 (90 mg/day) and 5 (40 mg/day) were treated with pyridoxine for nearly 4 months (at the last follow-up) without seizures at the age of four. With the two patients’ seizure frequency of once every 4–5 months, the efficacy of pyridoxine was difficult to assess.

Five patients showed severe developmental delay, particularly in language, while two patients were moderate. Patient 1 and 3 showed severe delayed intelligence and language with normal motor ability.

#### Phenotype of *PIGA* mutation

Among the eight male patients derived from 8 unrelated families (patient 8–15), patient 11 had a history of preterm labor and two patients showed perinatal abnormalities including anoxia or meconium-stained amniotic fluid. Patient 10 and 11 were diagnosed with congenital megacolon at the 28th days and the 13th days after birth, respectively. Convulsion occurred between 1 month and 28 days and 10 months. Focal seizures occurred in all patients. Other seizure types included PGTCS (1/8), GTCS (1/8), myoclonic seizure (1/8), absence seizure (1/8), and epileptic spasm (1/8), all of which could exist simultaneously in the same individual. In addition, three patients (patient 9, 10, and 11) had a history of status epilepticus. The first seizures in four patients occurred during a febrile episode, followed by febrile or afebrile seizures, mostly occurring during fever. The seizures of two patients showed a significant relationship with fever except for the first episode. The onset of seizures in patients 10 and 13 did not suggest a specific relationship with fever, but the seizures of patient 10 was aggravated during the course of febrile illness at times.

Physical examination data were not available in patient 15. In the remaining seven patients, three patients (patients 9, 10, and 11) showed facial dysmorphisms, with patient 9 also showing a foot deformity and an umbilical hernia. A cafe-au-lait-spot was observed on the back of patient 11. Except for two patients (patient 14 and 15) without available results, hypotonia could be seen in four patients. EEG findings were obtained from all eight patients. The initial interictal EEG showed hypsarrhythmia in patient 13. Other seven patients first demonstrated diffuse slow waves mixed with focal or multifocal discharges. Among the seven patients with brain MRI results, two patients were normal. While the most frequent abnormality was enlarged subarachnoid space (4/7), with others including enlarged ventricle (2/7), white-matter immaturity (1/7), and deeper brain sulcus (1/7). The above abnormalities could exist simultaneously in the same individual. Serum ALP level was mildly elevated (277 IU/L) in patient 10 at 6 months of age (normal range: 50–240 IU/L). As for patient 13, multiple results of serum ALP levels showed mild elevated (263 IU/L, normal range: 50–240 IU/L). The serum ALP levels of the other six patients were normal or without data.

Seizures of six patients were refractory to AEDs. In the other two patients, the seizures of patient 9 were controlled by the combination treatment of levetiracetam, valproic acid, and topiramate at the age of 16 months. Similarly, as for patient 14, the seizures were eventually controlled by valproate and levetiracetam at about 2 years of age. Three patients (patient 10, 11, and 13) were treated with pyridoxine. Among them, patient 10 and 11 were treated with pyridoxine intravenously for 6 days at the age of 6 months (100 mg/day) without effects, while patient 13 was treated with pyridoxine at the age of about 5 months, whose seizures remained uncontrolled no matter by the administration of oral (40 mg/day) or intravenous (100 mg/day). Besides, ketogenic diet was used in three patients during the course without obvious effects. All patients showed severe developmental delay, with patient 13 showing severe delayed intelligence and language with normal motor ability.

#### Phenotype of *PIGT* mutation

##### Patient 16

The male patient was the child of non-consanguineous Chinese parents. The first seizures occurred during fever at the age of 3, lasting for approximately 1 minute, with 7–8 times per year. Then the improved afebrile seizures occurred at the age of 9, uncontrolled by the combination of levetiracetam and oxcarbazepine. From the age of 9, he gradually became unsteady in walking and prone to falls with a worsen tendency. He is currently unable to stand up from sitting independently. Physical examination showed bilateral knee hyperreflexia, normal muscle strength and muscle tone, without facial or organ dysmorphism. EEG showed discharges in the left anterior and middle temporal areas during sleep at 9 years of age. After AEDs treatment, multiple EEG results were normal. Brain MRI and metabolic analyses in urine and plasma showed normal. Since the seizure onset, his motor development has been severely delayed, but intelligence and language are normal.

##### Patient 17

The male patient was the first child of non-consanguineous healthy Chinese parents. Pregnancy, labor and early infancy were normal. Motor milestones, like raising head and rolling over, were achieved later than infants of the same age. Seizures started at the age of 4 months, presenting as left lower limb shaking once or many times during the wake-up of 10–12 times per day. There was no significant correlation between seizures and fever. At about 5 months, the above seizures still existed without therapy, with the frequency increasing to 30–40 times per day. The seizures were reduced but not controlled by the therapy of valproate, levetiracetam, lamotrigine, and oxcarbazepine. Besides seizures, he showed startle responses to unexpected sound stimuli from childhood. Physical examination showed a cafe-au-lait-spot on his right knee. He had severe axial hypotonia, nasal bridge collapse, arched palatal, and dysplastic ears. At 10 months old, EEG showed multiple discharges and brain MRI showed enlarged subarachnoid space. With severe developmental delay, he was unable to stand or sit, having the unconscious pronunciation at 1 year old (the last follow-up).

### Molecular results

The details of molecular variants identified in *PIGN*, *PIGA*, and *PIGT* were summarized in Table 2, including the mutations of the genes with pedigrees, the frequency of variants among the normal population, and the scores in the prediction tool. And among the other variants, with only the *PIGA* mutation c. 1370C > T (p. A457V) predicted as “neutral” by SIFT, all the remaining were predicted to be “probably damaging”, “deleterious”, and “disease causing” to the protein structure identified by Polyphen-2, SIFT and Mutation Taster, respectively.

**Table 2:** Details of molecular variants identified in *PIGN*, *PIGA*, and *PIGT*

Patients ID	Mutation		Pathogenicity prediction			MAF datab			
	Mutation site	Parental Origin	Polyphen2	SIFT	Mutation Taster	ExAC	GnomAD	1000Genomes	dbSNP
**PIGN (NM_012327)**									
1	c.2122C > T(p.Q708X)	Paternal	Probably damaging	n.a.	Disease causing	/	/	/	/
	c.2557A > C(p.T853P)	Maternal	Probably damaging	Deleterious	Disease causing	/	/	/	/
2	c.2759_2760del(p.920fs)	Paternal	n.a.	n.a.	Disease causing	/	/	/	/
	c.1172 + 1G > A	Maternal	n.a.	n.a.	Disease causing	/	/	/	/
3	c.1109A > C(p.Q370P)	Paternal	Probably damaging	Deleterious	Disease causing	/	/	/	/
	c.694A > T(p.K232X)	Maternal	Probably damaging	n.a.	Disease causing	/	/	/	/
4	c.1694G > A(p.R565H)	Paternal	Probably damaging	Deleterious	Disease causing	0.000016	0.000032	0.0002	0.0002
	c.2663 T > C(p.I888T)	Paternal	Probably damaging	Deleterious	Disease causing	0.0001	/	/	/
	c.963G > A(p.Q321Q)	Maternal	n.a.	n.a.	Disease causing	0.0012	/	/	/
5	c.343G > C(p.G115R)	Paternal	Probably damaging	Deleterious	Disease causing	/	/	/	/
	c.1694G > T(p.R565L)	Maternal	Probably damaging	Deleterious	Disease causing	0.000016	000032	0.0002	/
6	c.505C > T(p.Q169X)	Paternal	Probably damaging	Deleterious	Disease causing	0	0	0	0
	c.769 T > G(p.F257V)	Maternal	Probably damaging	Deleterious	Disease causing	0.00011	0	0	0
7	c.895 C > T(p.Q299X)	Paternal	Probably damaging	Deleterious	Disease causing	/	/	/	0.000008
	c.629 T > C(p.L210S)	Maternal	Probably damaging	Deleterious	Disease causing	/	/	/	/
**PIGA (NM_002641)**									
8	c.356G > A(p.R119Q)	Maternal	Probably damaging	Deleterious	Disease causing	/	/	/	/
9	c.713A > G(p.K238R)	Maternal	Probably damaging	Deleterious	Disease causing	/	/	/	/
10	c.356G > A(p.R119Q)	Maternal	Probably damaging	Deleterious	Disease causing	/	/	/	/
11	c.241C > T(p.R81C)	Maternal	Probably damaging	Deleterious	Disease causing	/	/	/	/
12	c.929 T > A(p.L310H)	Maternal	Probably damaging	Deleterious	Disease causing	/	/	/	/
13	c.1370C > T(p.A457V)	Maternal	Probably damaging	Neutral	Disease causing	/	/	/	/
14	c.340A > T(p.R114W)	Maternal	Probably damaging	Deleterious	Disease causing	/	/	/	/
15	c.166C > T(p.L56F)	De novo	Probably damaging	Deleterious	Disease causing	/	/	/	/
**PIGT (NM_015937)**									
16	c.469 T > G(p.F157V)	Paternal	Probably damaging	Deleterious	Disease causing	/	/	/	/
	c.1579_1581delinsCAT(N527H)	Paternal	Probably damaging	Deleterious	Disease causing	/	/	/	/
	c.1120A > G(p.N374D)	Maternal	Probably damaging	Deleterious	Disease causing	/	/	/	/
17	c.514C > T(p.R172C)	Paternal	Probably damaging	Deleterious	Disease causing	0.000008	0.00004	/	/
	c.98delA(p.E33Dfs*11)	Maternal	n.a.	Deleterious	Disease causing	/	/	/	/

*MAF* minor allele frequency, *ExAC* exome aggregation consortium, *GnomAD* genome aggregation database, *dbSNP* database of single nucleotide polymorphism, *n.a.* not available, / not included

#### *PIGN* mutations

Fifteen variants from seven patients were identified with *PIGN* mutations (NM_012327), including 8 missense mutations, 4 nonsense mutations (one variant has been reported) [16], one synonymous mutation (has been reported) [15], one splicing site mutation, and one deletion mutation. Among all patients, case 4 had 3 mutations. Two mutations [c.694A > T (p. K232X) and c.963G > A (p. Q321Q)] had been reported previously [15, 16]. All variants were validated by Sanger sequencing confirming their parental inheritance.

#### *PIGA* mutations

Mutation analysis identified seven different hemizygous *PIGA* mutations (NM_002641) among eight patients, all of which were missense mutations. Of them, R119Q presented in two patients here and had been reported before [34]. Seven of the mutations were inherited from their mothers, and only one was de novo.

#### *PIGT* mutations

Five *PIGT* variants were identified in two sporadic patients (NM_015937), while case 16 carried 3 mutations. Among these, three mutations were missense mutations, while the other two were deletion mutation and deletion insertion mutation. None of the mutations had been previously reported. The c.469 T > G variant was a missense transition from phenylalanine to valine. Additionally, it was predicted to be deleterious by three algorithms (PolyPhen-2, SIFT, and Mutation Taster). The c.1120A > G variant was also predicted to be highly damaged to the protein structure. The c.514C > T variant is a missense transition from arginine to cysteine at residue 172 of the protein, predicted as deleterious by the above predictive software packages.

## Discussion

In 17 patients with a congenital disorder of MCAHS, the whole exome sequencing revealed pathogenic variants of the *PIGN*, *PIGA*, and *PIGT*. Among them, one patient had 3 *PIGN* mutations, and another patient had 3 *PIGT* mutations. There was no previous report of patients with 3 *PIGN*, *PIGA*, or *PIGT* mutations simultaneously. In the wide variation of the MCAHS phenotypic spectrum, our patients differed significantly in addition to the several similarities with the previously reported cases, broadening the scope of knowledge on inherited GPI deficiency.

Early onset seizures appeared to be a common feature of the 83 patients previously reported with *PIGN*, *PIGA*, and *PIGT* mutations. Based on previously reported cases, the most common type in patients with *PIGN* mutations [5, 9–19] was focal seizure, which also presented in our patients. Three patients with *PIGN* mutations showed atypical absence, which had not been seen in previous reports. By summarizing the *PIGA* mutations cases reported, myoclonic seizure was the most common type (10/19 patients with available data) [6, 22–34]. However, Tarailo-Graovac et al. [24] who described one case and summarized 10 cases reported previously found that infantile spasms with hypsarrhythmia on EEG was the hallmarks of these patients. Different from the above reports, our eight cases with *PIGA* mutations were characterized by neither epileptic spasms nor myoclonic seizures, but by focal seizures (100%, 8/8) and status epilepticus (38%, 3/8). Besides, EEGs, were more of diffuse slow waves mixed with focal or multifocal discharges (7/8), but did not reach the degree of hypsarrhythmia. In the previous reports, myoclonic seizure (17/25 patients with available data) was the most common type in *PIGT* mutations patients, without epileptic spasms presented [7, 36–43]. In our cohort, focal seizures, epileptic spasms, and myoclonic seizures occurred in both patients with *PIGT* mutations.

Intractable seizures occurred in the majority reported previously (60/72 patients with available data) and here [5–7, 9–19, 22–34, 36–43]. Chiyonobu et al. [44] hypothesized that the seizures in these patients were due to a defect in intraneuronal pyridoxal phosphate (PLP), a cofactor of γ-amino butylic acid (GABA) synthase, which was caused by a loss of membrane-anchored ALP, leading to a shortage of GABA. Pyridoxine treatment was reported to be effective for seizures in some patients with inherited GPI deficiencies, such as *PIGV* or *PIGO* mutations [45–47]. However, two patients with *PIGN* mutations and three patients with *PIGA* mutations in this study failed to respond to pyridoxine treatment. Up to now, only two patients with *PIGA* mutations and one patient with *PIGT* mutations were reported to receive pyridoxine treatment [30, 34, 40]. In addition, two patients with *PIGT* mutations have also been treated with PLP [36, 40]. Neither pyridoxine nor PLP were effective, although the number of cases was limited. It was unclear whether genetic heterogeneity played a role in only some individuals’ responsiveness to pyridoxine but not in others. Therefore, more patients with intractable seizures should be treated with pyridoxine or PLP to determine whether such treatment might be effective for some patients with *PIGN*, *PIGA*, and *PIGT* mutations.

In terms of development, among previously reported cases, except for two patients with moderate delay carrying *PIGN* and *PIGA* mutations [17, 30], the others had severe delay or early death [5–7, 9–19, 22–34, 36–43]. In our cohort, there are two patients with moderate delay carrying *PIGN* mutations. In addition, patient 16 with *PIGT* mutations showed mild developmental delay without dysmorphisms or severe intellectual disability. Moreover, the onset of seizures was relatively late, and the findings of brain MRI were normal. To date, he was the only *PIGT* mutations patient having neither facial nor other organ defects with the latest seizure onset age. Overall, the clinical phenotype of patient 16 expanded the clinical presentation of MCAHS3.

Hyperphosphatemia is a characteristic symptom of some GPI deficiencies, including *PIGO*, *PIGW*, *PGAP2*, etc. [48–50]. Such elevations have not been reported in the previous cases of *PIGN* mutations. However, patient 4 with 3 *PIGN* mutations, showed high serum ALP levels. There were no previous reports of patients with 3 *PIGN* mutations simultaneously which might have a great influence on the function of GPI. Therefore, the increased level of serum ALP in patient 4 might be caused by the dysfunction of GPI protein due to the insufficient residual function of *PIGN*, based on the mechanism of *PIGA* mutations. Mildly elevated serum ALP was also reported in more than half of the patients with *PIGA* mutations (9/15 patients with available data), including two patients here [6, 22–34]. A possible biological explanation was that when cell surface GPI-APs, such as ALP, could not be modified by GPI, they were secreted as soluble non-GPI-anchored proteins [50]. The elevated serum ALP might serve as a diagnostic clue rather than a criterion. In contrast, hypophosphatasia was a particularly distinctive feature in patients with *PIGT* mutations (7/13 patients with available data) [7, 36–43]. Murakami et al. [50] considered that abnormal GPI transaminase resulted in the difficulty in hydrolysis of ALP precursor protein, leading to the degradation of most of the precursor protein in cells and the decrease of serum ALP. However, the feature of hypophosphatasia was not found in our two *PIGT* mutations cases. In addition, the level of GPI anchored proteins on the granulocyte surface decreased in patients with GPI deficiencies, such as CDl6, CD24, CD59, etc. [5, 15]. In this study, flow cytometry was performed on only one case with *PIGN* mutations. The result showed that the expression levels of total GPI-APs CD16, CD24, and CD59 in peripheral blood granulocytes were decreased, which confirmed the effect of mutation on *PIGN* function.

By summarizing the previously reported cases, in patients with *PIGN* mutations, white matter immaturity and thinning of the corpus callosum were the most common brain MRI abnormities (38%, 8/21 patients with available data) [5, 9–19]. Brain MRI showed cerebellar atrophy in 76% of the *PIGT*-deficient patients (10/14 patients with available data), which also accounted for the majority of patients with *PIGA* mutations (25%, 4/16 patients with available data) [6, 7, 22–34, 36–43]. Different from the previous reports, enlarged subarachnoid space was the most common brain MRI abnormality in the patients we reported, regardless of which type of mutations.

Up to now, different *PIGN*, *PIGA*, and *PIGT* disease-causing variants have been reported in 22, 15, and 15 mutation sites, respectively [5–7, 9–19, 22–34, 36–43]. Here, we identified 15 *PIGN* variants (one patient carrying 3 mutations), 8 *PIGA* variants, and 5 *PIGT* variants (one patient carrying 3 mutations). In the case of patient 4 with *PIGN* mutations, there were two paternal mutations and one maternal. The pathogenicity of the maternal synonymous mutation site c.963G > A has been reported and proved to have actual effects of on splicing [15]. The presence of 3 mutations all affected the function of GPI-APs and made the phenotype of patient 4 more severe, which was characterized by a history of preterm labor, intractable seizures, severe developmental delay, multiple facial and other organ deformities, and especially elevated serum ALP level. Previous reports had suggested a genotype-phenotype correlation of *PIGN* mutations [14, 18]. The clinical severity of the cases reported to date seemed to correlate with the predicted functional severity of the mutations in *PIGN* [14]. Specifically, the fatal biallelic truncating variant of *PIGN* was identified in patients with a severe clinical course, including early onset of intractable seizures and death in utero or shortly after birth. Our patients did not carry biallelic loss of function variants and showed the milder phenotype without major visceral congenital anomalies. It was further proved that the biallelic mutations predicting truncated proteins with presumed loss of function variants were likely responsible for the severe phenotype of *PIGN* mutations.

As for the variants of *PIGA*, to date, a total of seven patients carrying c.1234C > T have been reported, with a very similar phenotype [6, 21, 29]. This suggested that the phenotype might correlate with genotype and the residual functional activity of the *PIGA* protein. However, though our two patients with the same c.356G > A mutation had similar onset of seizures, AEDs resistance, and severe developmental delay, multiple organ anomalies and hypotonia only occurred in one of them. This variability indicated that the phenotype was influenced by other factors in addition to genotypes. In general, the phenotypic spectrum caused by *PIGA* germline mutations were classified into two types previously, severe and less severe [30]. According to phenotype and genotype, 16 out of 25 patients reported previously were divided into the severe group, as was our patient 10 [6, 22–34]. However, in the rest of the patients, only a few (Patient IV-2 by Belet et al., 2013; patient 3 and patient 4 by Kato et al., 2014; patient 1 and patient 2 by Joshi et al., 2016) characteristic of the less severe form [26, 27, 30]. The remaining 11 patients reported here, and previously, their phenotypes could hardly be divided into severe or less severe group. For example, except for patient 10, though the other seven patients reported here presented with intractable seizures (5/7), severe developmental delay (7/7), multiple organ malformations (2/6), hypotonia (3/5), lack of dysmorphism, elevated serum ALP levels precluded them to be included into severe form. And obviously, they did not belong to the less severe group. It indicated that the phenotypic range of *PIGA* mutations was more continuous than discrete.

Additionally, previous reports had shown that *PIGT* was expressed at the highest level in the central nervous system, which might explicate that the neurological defect was the core features and the most severe [38, 39]. Abnormalities in other organ systems might not be considered as a necessary diagnostic factor for *PIGT* mutations [38, 39]. However, mild neurological symptoms and developmental status were detected in our proband (patient 16), with motor ability decreasing after seizures onset. In addition to the two missense mutations, patient 16 also carried a paternal deletion insertion mutation, both of which involved three bases. Thus, each of the three mutations altered a single amino acid in GPI-APs and was predicted by three algorithms (PolyPhen-2, SIFT, and Mutation Taster) to be pathogenic. One possible explanation for its lighter phenotype was that amino acids altered by *PIGT* mutations had less effect on GPI-APs function. Moreover, whether these three mutations or one of them would always be related to the milder phenotype needs more cases to be verified. In short, *PIGT* mutations could also cause mild neurological manifestations without other malformations.

## Conclusions

We expand our knowledge of MCAHS in terms of phenotype and genotype. There were 7 patients with 15 *PIGN* mutations, 8 patients with 8 *PIGA* mutations, and 2 patients with 5 *PIGT* mutations. All patients had epilepsy and developmental delay, and 71% of them showed hypotonia. Focal seizure was the most common type in all cases. Eighty-two percent of patients showed a significant relationship between seizures and fever. Brain MRI abnormalities showed enlarged subarachnoid space in 56% patients. Among patients with *PIGN* mutations, atypical absence seizures had not been documented in previous reports. We have described here the only patient carrying *PIGN* mutations with elevated serum ALP. Our cases with *PIGA* mutations were characterized by focal seizures with diffuse slow waves mixed with focal or multifocal discharges on EEG rather than infantile spasms with hypsarrhythmia [24]. Except for one with *PIGA* mutations classified as severe, it was difficult to classify the phenotypes of our remaining seven patients as severe or less severe group, which indicated that phenotypes were more continuous than discrete. Mild neurological symptoms and developmental status of one patient with *PIGT* mutations expanded the clinical presentation of MCAHS3.

## Data Availability

The datasets generated and/or analyzed during the current study are not publicly available due to patient data safety restrictions but are available from the corresponding author on reasonable request.

## Abbreviations

GPI: Glycosylphosphatidylinositol
GPI-APs: GPI-anchored proteins
MCAHS: Multiple congenital anomalies-hypotonia-seizures syndrome
PIGN: Phosphatidylinositol glycan biosynthesis class N protein
PIGA: Phosphatidylinositol glycan biosynthesis class A protein
PIGT: Phosphatidylinositol glycan biosynthesis class T protein
PGTCS: Partial secondarily generalized tonic-clonic seizure
GTCS: Generalized tonic-clonic seizure
EEG: Electroencephalogram
MRI: Magnetic resonance imaging
AEDs: Antiepileptic drugs
ALP: Alkaline phosphatase
PLP: Pyridoxal phosphate
GABA: γ-amino butylic acid
CDH: Congenital diaphragmatic hernia
